# Novel Giomers Incorporated with Antibacterial Quaternary Ammonium Monomers to Inhibit Secondary Caries

**DOI:** 10.3390/pathogens11050578

**Published:** 2022-05-14

**Authors:** Yandi Chen, Bina Yang, Lei Cheng, Hockin H. K. Xu, Hao Li, Yuyao Huang, Qiong Zhang, Xuedong Zhou, Jingou Liang, Jing Zou

**Affiliations:** 1 State Key Laboratory of Oral Diseases, National Clinical Research Center for Oral Diseases, West China School of Stomatology, Sichuan University, Chengdu 610041, China; chenyandi@stu.scu.edu.cn (Y.C.); 2019224035115@stu.scu.edu.cn (B.Y.); chenglei@scu.edu.cn (L.C.); 2019324030005@stu.scu.edu.cn (H.L.); 2014181641009@stu.scu.edu.cn (Y.H.); qiongzhang@scu.edu.cn (Q.Z.); zhouxd@scu.edu.cn (X.Z.); 2 Department of Pediatric Dentistry, West China School of Stomatology, Sichuan University, Chengdu 610041, China; 3 Department of Cariology and Endodontics, West China School of Stomatology, Sichuan University, Chengdu 610041, China; 4 Department of Advanced Oral Sciences and Therapeutics, School of Dentistry, University of Maryland, Baltimore, MD 21201, USA; hxu@umaryland.edu; 5 Center for Stem Cell Biology and Regenerative Medicine, School of Medicine, University of Maryland, Baltimore, MD 21201, USA; 6 Marlene and Stewart Greenebaum Cancer Center, School of Medicine, University of Maryland, Baltimore, MD 21201, USA

**Keywords:** early childhood caries, secondary caries, biofilms, giomers, antibacterial quaternary ammonium monomers

## Abstract

The objective of this study was to develop novel modified giomers by incorporating the antibacterial quaternary ammonium monomers (QAMs), dimethylaminododecyl methacrylate (DMADDM) or dimethylaminohexadecyl methacrylate (DMAHDM) into a commercial giomer. The material performances including mechanical properties, surface characteristics, color data, cytotoxicity and fluoride release of the novel giomers were evaluated. Antibacterial activity against severe early childhood caries (S-ECC) saliva-derived biofilms was assessed by lactic acid production measurement, MTT assay, biofilm staining and 16S rRNA sequencing. A rat model was developed and the anti-caries effect was investigated by micro-CT scanning and modified Keyes’ scoring. The results showed that the material properties of the QAMs groups were comparable to those of the control group. The novel giomers significantly inhibited lactic acid production and biofilm viability of S-ECC saliva-derived biofilms. Furthermore, caries-related genera such as *Streptococcus* and *Lactobacillus* reduced in QAMs groups, which showed their potential to change the microbial compositions. In the rat model, lesion depth, mineral loss and scoring of the QAMs groups were significantly reduced, without side effects on oral tissues. In conclusion, the novel giomers incorporated with antibacterial QAMs could inhibit the cariogenic biofilms and help prevent secondary caries, with great potential for future application in restorative treatment.

## 1. Introduction

Early childhood caries (ECC) is a health problem worldwide, and can severely affect children’s oral and systemic health. It has been reported that 48% of overall preschool children are affected by ECC, and currently the prevalence in China is 71.9% among five-year-olds [1,2]. ECC may impair children’s masticatory function, influence pronunciation, induce facial esthetic problems, and increase the risk of caries and malocclusion in permanent dentition [2,3,4].

A common treatment against ECC is to remove the carious lesions and place dental restorations. However, even after restorative treatment, recurrences of lesions at the margins of existing restorations, known as secondary caries, are commonly reported [5,6]. Children with many risk factors are particularly susceptible to secondary caries [7]. The most significant reasons for ECC in young children are poor oral hygiene and excessive dietary sugar intake. These habits are conducive to the accumulation and growth of cariogenic biofilms [5,7,8,9]. Furthermore, placing restorations for children at young ages is challenging due to limited cooperation, which consequently leads to a rise in secondary caries [7,10]. Once secondary caries occur, retreatment and additional dental tissue removal are usually required [11]. Currently, the limited ability of conventional restorative materials in inhibiting secondary caries is still a problem [7]. Thus, developing novel anti-caries restorative materials for children is of great importance.

Giomers have been widely used in restorative treatment against ECC on account of their excellent mechanical, esthetic and handling properties [12,13]. They are hybrid materials containing pre-reacted glass fillers in the resin matrix, thus possessing the ability to release fluoride [13]. However, the fluoride release and recharge levels of giomers were not enough to inhibit the formation and acid production of biofilms [14,15,16]. Therefore, it is beneficial to improve the antibacterial effects of giomers in order to inhibit cariogenic biofilms and better prevent secondary caries in children.

Many attempts have been made to improve the antibacterial activity of restorative materials. One was the incorporation of antibacterial agents, especially immobilized agents. Quaternary ammonium monomers (QAMs) are cationic compounds showing “contact-killing” effects [17,18]. QAMs such as dimethylaminododecyl methacrylate (DMADDM) and dimethylaminohexadecyl methacrylate (DMAHDM) are able to co-polymerize with the resin matrix by the methacrylate groups, and exhibit non-releasing and long-term antimicrobial effects [18,19,20] (the molecular structures of DMADDM and DMAHDM were shown in Supplementary Material Figure S1). They have been incorporated into dental materials such as composites, adhesives and denture resins. The QAMs-modified materials showed enhanced anti-biofilm activity with comparable mechanical properties and biocompatibility [17,21,22,23,24]. To our knowledge, very few studies have been done on the modification of giomers by antibacterial agents. In particular, no previous study has investigated the QAMs-modified giomers on their potential in the treatment and prevention for ECC.

In this study, we investigated the potential of QAMs-modified giomers for the prevention of secondary caries in children. Novel giomers incorporated with DMADDM and DMAHDM were developed, and a severe early childhood caries (S-ECC) saliva-derived biofilm model was established to imitate the cariogenic biofilms in children. Our hypothesis was that the novel modified giomers had a significant antibacterial effect and could inhibit the secondary caries, with good material performances and biocompatibility in oral tissues. They also had the potential to change the compositions of the cariogenic biofilms.

## 2. Results

### 2.1. Mechanical Properties

**Figure 1:**
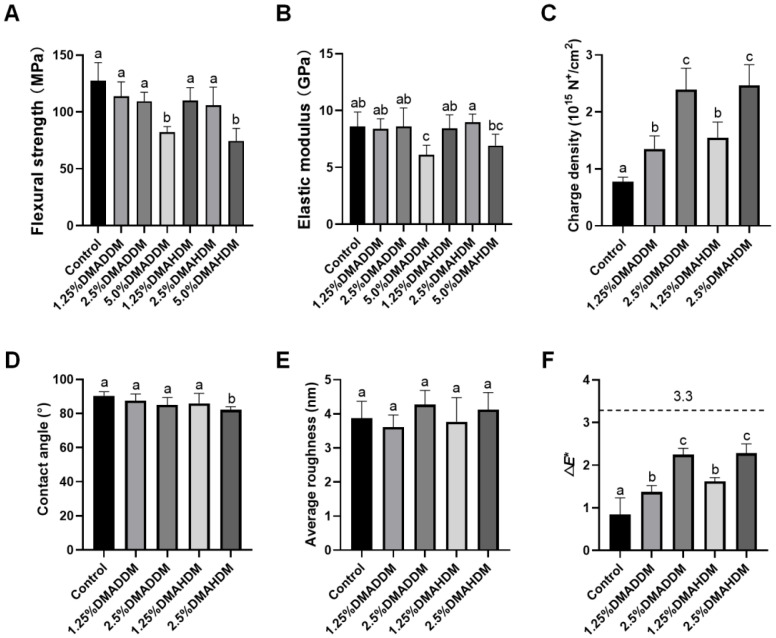
Material performances. (**A**) Flexural strength. (**B**) Elastic modulus. (**C**) Surface charge density. (**D**) Contact angle. (**E**) Surface roughness. (**F**) Color change (ΔE*) between each group and the control group. Data are presented as mean ± SD; *n* = 6. In each plot, bars with the same letter(s) indicate no significant difference between the groups (*p* > 0.05), while bars without the same letter(s) indicate significant difference between the groups (*p* < 0.05).

The results of flexural strength and elastic modulus are shown in Figure 1A,B. No statistically significant difference in either flexural strength or elastic modulus was discovered after incorporation of 1.25% or 2.5% DMADDM/DMAHDM (*p* > 0.05). Furthermore, the flexural strength of each specimen in the 1.25% and 2.5% QAMs groups was higher than 80 MPa, a minimal value according to the criteria of ISO 4049/2000 [25]. However, significantly reduced flexural strength and elastic modulus were discovered in the 5.0% DMADDM group (*p* < 0.05). The 5.0% DMAHDM group also showed reduced flexural strength (*p* < 0.05).

### 2.2. Surface Characteristics

The surface charge density, contact angle and average roughness of each group are presented in Figure 1C–E. After the incorporation of DMADDM/DMAHDM, significantly higher surface charge densities were observed (*p* < 0.05). The contact angle values were slightly lower in the 2.5%DMAHDM group (*p* < 0.05). The representative images of contact angles are shown in Figure S2. In addition, no significant difference in surface roughness was found between each QAMs group and the control group (*p* > 0.05). The representative 3D images are shown in Figure S3.

### 2.3. Colorimetric Analysis

The color data and a three-dimensional scatter diagram are presented in Figure S4. Increased L* values were observed in two DMAHDM groups (*p* < 0.05). Increased a* values were observed in the 2.5% DMADDM, 1.25% DMAHDM and 2.5% DMAHDM group (*p* < 0.05). Furthermore, b* values decreased in the 1.25% DMADDM, 2.5% DMADDM and 2.5% DMAHDM groups (*p* < 0.05). The color difference between each QAMs group and the control group was statistically significant (*p* < 0.05), as shown in Figure 1F. However, all samples were considered to be clinically acceptable (ΔE* < 3.3) [26].

### 2.4. Cytotoxicity Test of the Eluants

The cytotoxicity test of the eluants at each concentration is demonstrated in Figure S5. The results showed no significant differences in cell viability in all groups (*p* > 0.05).

### 2.5. Fluoride Release and Recharge

**Figure 2:**
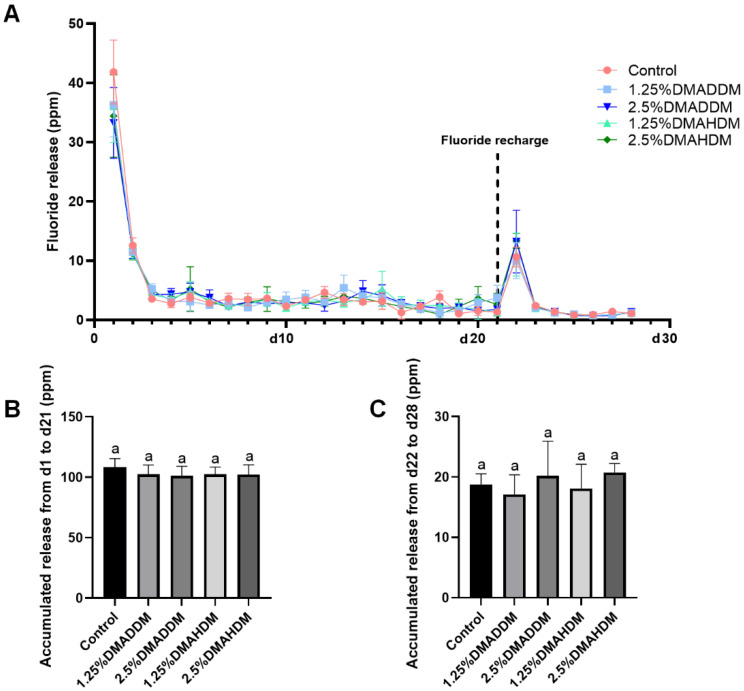
Fluoride release measurement. (**A**) The daily fluoride release curves. (**B**) Accumulation of initial fluoride release from d1 to d21. (**C**) Accumulation of fluoride re-release from d22 to d28. Data are presented as mean ± SD; *n* = 6. In plot (**B**,**C**), bars with the same letter indicate no significant difference between the groups (*p* > 0.05).

The initial fluoride release (d1–d21) and re-release (d22–d28) are presented in Figure 2. All groups exhibited similar ion release patterns (Figure 2A). The accumulated ion release either during d1–d21 (Figure 2B) or during d22–d28 (Figure 2C) was comparable in all groups (*p* > 0.05).

### 2.6. Antibacterial Activity

**Figure 3:**
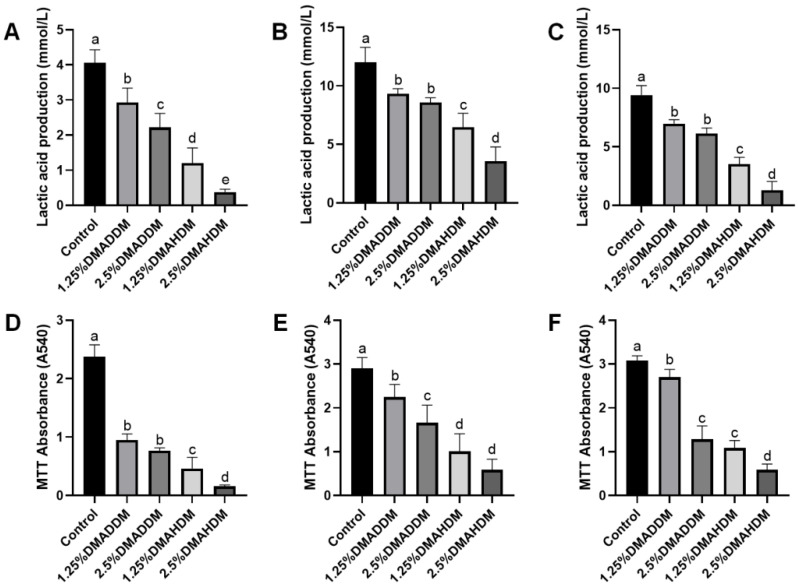
Antibacterial activity of giomers. (**A**–**C**) Lactic acid production in 24 h (**A**), 48 h (**B**) and 72 h (**C**) biofilms. (**D**–**F**) MTT absorbance in 24 h (**D**), 48 h (**E**) and 72 h (**F**) biofilms. Data are presented as mean ± SD; *n* = 6. In each plot, bars with the same letter indicate no significant difference between the groups (*p* > 0.05), while bars with different letters indicate significant difference between the groups (*p* < 0.05).

Lactic acid production and MTT assay results at 24 h, 48 h and 72 h of incubation are shown in Figure 3. The lactic acid production and the biofilm metabolic activity at each time period significantly reduced after incorporation of 1.25% or 2.5% QAMs (*p* < 0.05). The lactic acid production and biofilm metabolic activities were lower in the DMAHDM groups than in the DMADDM groups at the same concentrations of QAMs (*p* < 0.05).

**Figure 4:**
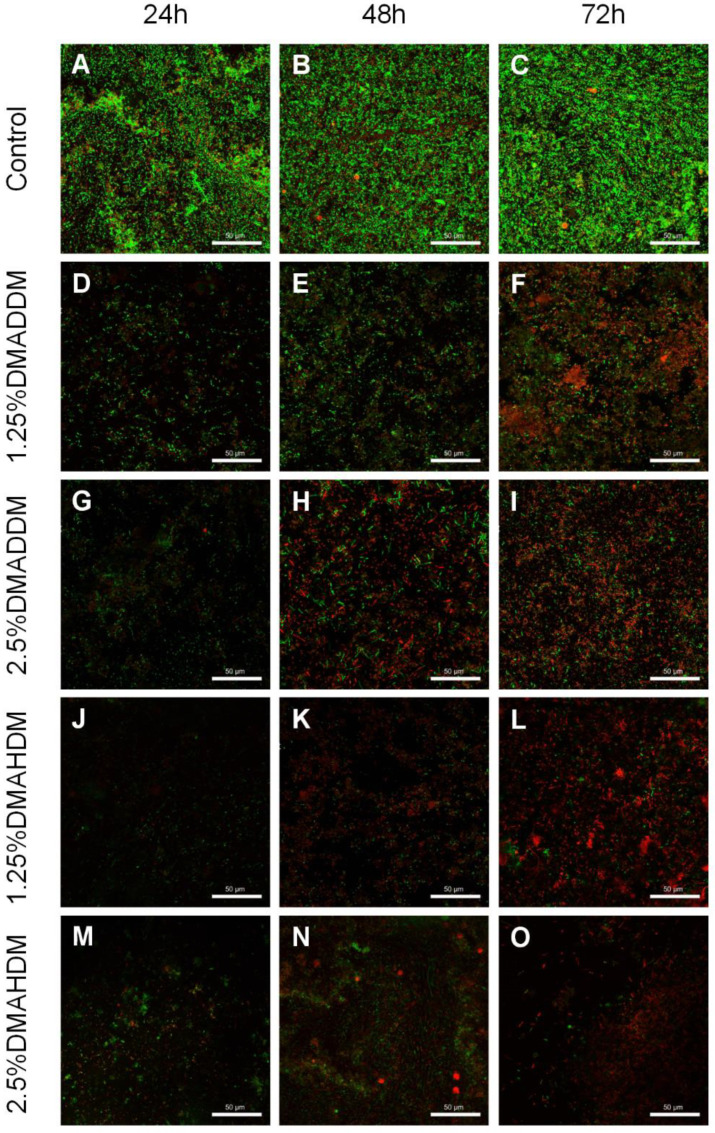
Representative live/dead staining images of S-ECC saliva-derived biofilms. (**A**–**C**) the control, (**D**–**F**) 1.25% DMADDM, (**G**–**I**) 2.5% DMADDM, (**J**–**L**) 1.25% DMAHDM and (**M**–**O**) 2.5% DMAHDM. Scale bars = 50 μm.

The live/dead staining images (Figure 4) revealed that in the control group, the biofilm growth and viability were very high since the bacteria were mostly stained green. In the QAMs groups, biofilm formation was reduced and most of the bacteria were stained red.

**Figure 5:**
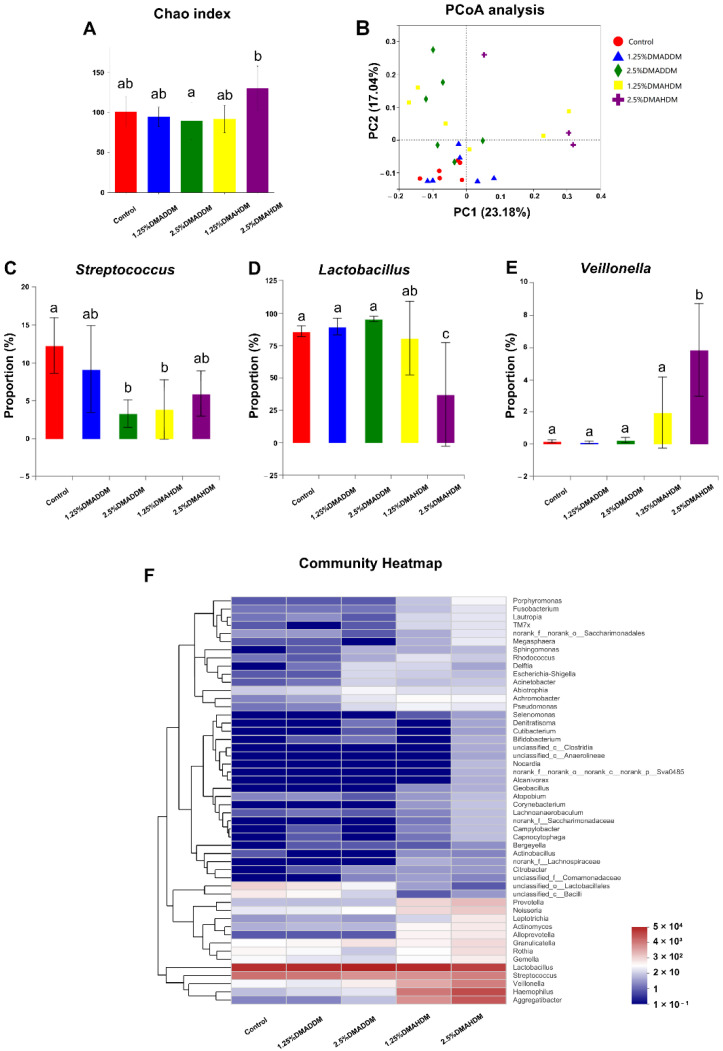
16S rRNA sequencing results. (**A**) Chao index. (**B**) PCoA analysis. (**C**–**E**) Proportions of *Streptococcus* (**C**), *Lactobacillus* (**D**) and *Veillonella* (**E**). Data are presented as mean ± SD; n = 6. In each plot, bars with the same letter(s) indicate no significant difference between the groups (*p* > 0.05), while bars without the same letter(s) indicate significant difference between the groups (*p* < 0.05). (**F**) Community heatmap at genus level.

To investigate the compositions of the biofilms, we used 16S rRNA sequencing to analyse the 72 h-biofilms. The rarefaction curves reached the plateaus (Figure S6A) and a total of 116, 129, 156, 170, 173 OTUs were found in the control group, 1.25% DMADDM group, 2.5% DMADDM group, 1.25% DMAHDM group and 2.5% DMAHDM group respectively (Figure S6B). There was no significant difference in Chao index between each QAMs group and the control group (Figure 5A). Figure 5F shows the community heatmap of the biofilms in each group. The PCoA analysis (Figure 5B) demonstrated that the 1.25% DMADDM group nearly clustered with the control group, while other QAMs groups were separated from the control group. There were three genera in varied proportions among groups, as displayed in Figure 5C–E. The 1.25% DMADDM group showed comparable levels of *Streptococcus*, *Lactobacillus*, and *Veillonella* to the control group. In the 2.5% DMADDM and 1.25% DMAHDM groups, levels of *Lactobacillus* and *Veillonella* were comparable to those in the control group, while *Streptococcus* was less abundant than in the control group (*p* < 0.01). The 2.5% DMAHDM group showed a lower level of *Lactobacillus* (*p* < 0.001) but a higher level of *Veillonella* than the control group (*p* < 0.01). The percentage of *Streptococcus* in the 2.5% DMAHDM group was lower than that in the control group, but not statistically significant (*p* > 0.05). It is noteworthy that *Aggregatibacter* and *Haemophilus* were detected in high proportions in the DMAHDM groups, but hardly found in other groups, as shown in Figure S6C,D.

### 2.7. Secondary Caries in a Rat Model

**Figure 6:**
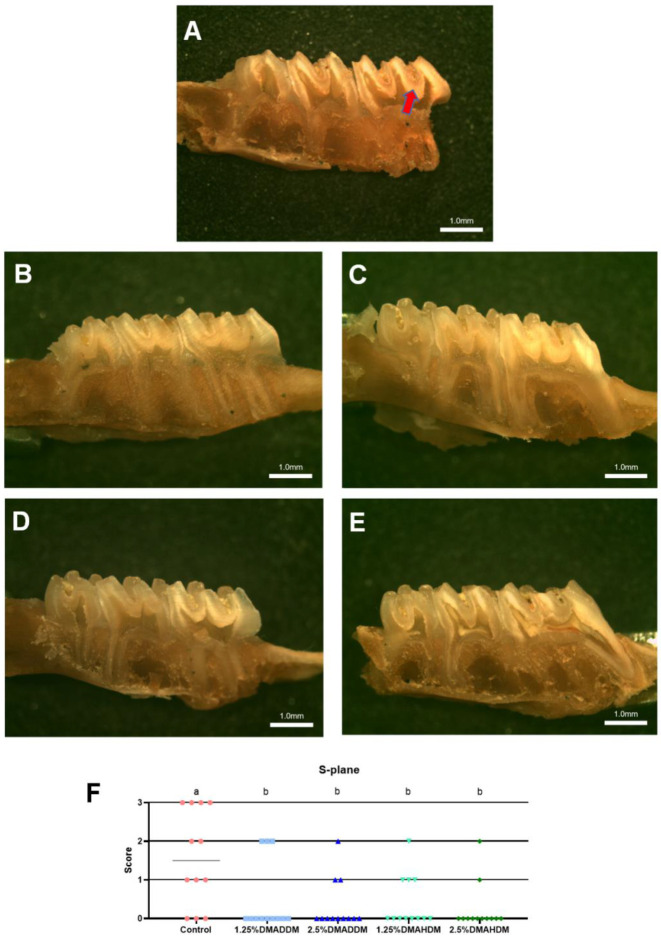

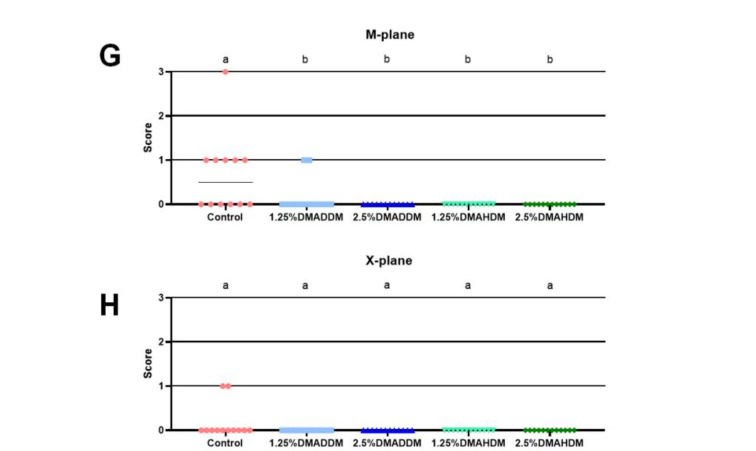
Evaluation of secondary caries by modified Keyes’ scoring. (**A**–**E**) Representative images of maxillary specimens: the control (**A**), 1.25% DMADDM (**B**), 2.5% DMADDM (**C**), 1.25% DMAHDM (**D**) and 2.5% DMAHDM (**E**). The carious lesions were dyed red (red arrow). Scale bars = 1 mm. (**F**–**H**) Modified Keyes’ scoring of the lesions in the S-plane (**F**), M-plane (**G**) and X-plane (**H**). Data are presented as scatter plots; *n* = 12. In each plot, groups with the same letter indicate no significant difference between them (*p* > 0.05), while groups with different letters indicate significant difference between them (*p* < 0.05).

**Figure 7:**
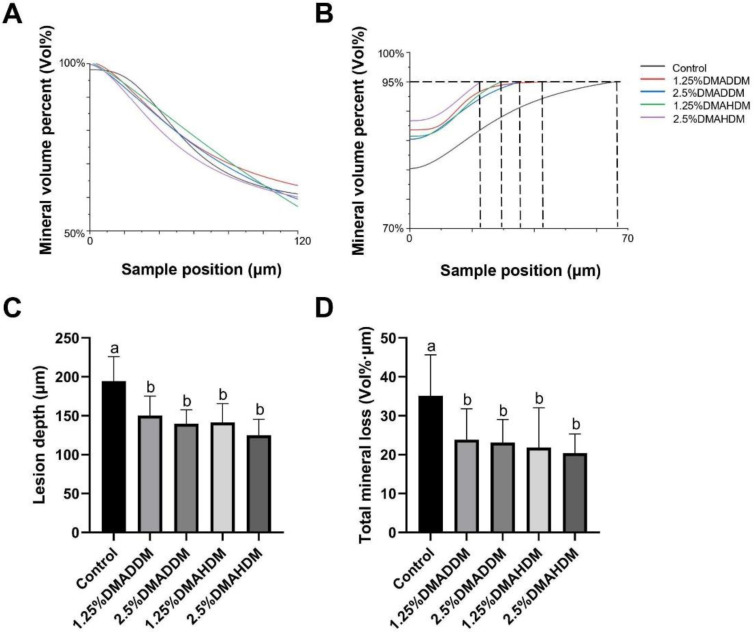
Evaluation of secondary caries by micro-CT. (**A**,**B**) Mineral volume curves of enamel (**A**) and dentin (**B**). (**C**) Lesion depth in rat teeth. (**D**) Total mineral loss in rat teeth. Data are presented as mean ± SD; *n* = 12. In plot (**C**,**D**), bars with the same letter indicate no significant difference between the groups (*p* > 0.05) while bars with different letters indicate significant difference between the groups (*p* < 0.05).

HE staining images of the soft tissues are shown in Figure S7. No evident inflammatory response was observed in all groups. The representative images of maxillary specimens are demonstrated in Figure 6A–E. The modified Keyes’ scores of the lesions are presented in Figure 6F–H. In the S-plane and M-plane, the scoring method showed significantly lower scores in the QAMs groups (*p* < 0.05). In the X-plane, two samples from the control group were scored “1”, and all QAMs groups had scores of “0”, but no significant difference was discovered in all groups (*p* > 0.05). The micro-CT measurement demonstrated that in the mineral volume curves of enamel and dentin, all groups had similar trends (Figure 7A,B). The lesion depth and total mineral loss in the QAMs groups were significantly lower than those in the control group (Figure 7C,D), *p* < 0.05).

## 3. Discussion

With an increasing burden of ECC worldwide and high failure rate of restorative treatment, there is a need for new restorative materials with enhanced antibacterial performance that can effectively inhibit secondary caries in children. In the present study, we incorporated two QAMs, viz. DMADDM and DMAHDM, into conventional giomers to obtain novel antibacterial giomers. We expected that these QAMs-modified giomers would be appropriate for the treatment of ECC.

The investigation on the material performances demonstrated that incorporation of 1.25% or 2.5% DMADDM/DMAHDM did not influence the mechanical properties such as flexural strength and the elastic modulus of the giomers. It has been concluded in the previous studies that incorporating QAMs in proper proportions did not impair the mechanical properties of dental materials, and that DMADDM and DMAHDM, which contained methacrylate groups in their molecules, could be co-polymerized to the resin matrix [21,27,28]. However, in the present study, significantly reduced flexural strength in the 5.0% QAMs groups and reduced elastic modulus in the 5.0% DMADDM group were observed. Thus, considering that improvements of giomers should not impair the mechanical properties of the parent materials, the 5.0% QAMs groups were excluded from the following studies.

The pathogenic process of caries is primarily dependent on the formation of cariogenic biofilms [5,29]. The surface characteristics are critical in bacterial adhesion. As reflected in the surface charge test, the surfaces of the novel giomers were adequately positively-charged after incorporation of the cationic QAMs which had been chemically bonded to the resin matrix of the giomers, thus showing stronger antimicrobial potency [21,30]. Meanwhile, their roughness was comparable to the commercial control. We also discovered that adding QAMs slightly influenced the wettability of the parent giomers. As can be observed from Figure 1D, the hydrophilicity increased in the 2.5% DMAHDM group. However, all contact angles were greater than 65°, indicating that all specimens could be considered as very hydrophobic [31]. Although some studies concluded that bacteria preferred to adhere to more hydrophilic surfaces [32,33], diminished bacterial adhesion on more hydrophobic surfaces was not reported in some other studies [34,35,36]. In this study, reduced biofilm formation was observed on the 2.5% DMAHDM-modified giomer specimens (Figure 4M–O). On the contrary, thick and extensive biofilms were observed on the commercial control (Figure 4A–C). We speculated that under the present experimental conditions, biofilm adhesion was mainly influenced by the antibacterial activity.

**Figure 8:**
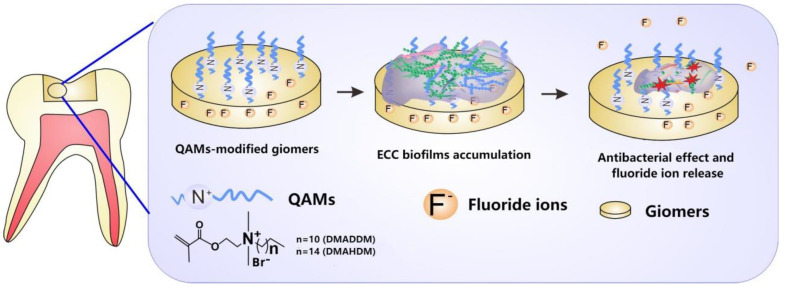
Schematic illustration of the QAMs-modified giomers with non-releasing antibacterial effect and fluoride release property.

By means of colorimetric analysis, we found that the overall color changes in all QAMs groups were clinically acceptable according to a boundary value of 3.3 [26]. The cytotoxicity test revealed that the QAMs-modified giomers exhibited good biosafety. More prominently, the QAMs-modified giomers not only showed excellent non-releasing antibacterial potency, but also well preserved fluoride release and recharge functions. This is of importance because fluoride is effective in arresting and remineralizing carious lesions [37]. Clinically, a restoration with such double anti-caries effects would be very prominent (Figure 8).

To investigate the antibacterial effect of the QAMs-modified giomers, and to provide an in-vitro model that can effectively simulate cariogenic biofilms of ECC, an S-ECC saliva-derived biofilm model was originally established. The biofilms were incubated on tested specimens in the SHI medium, which was reported to be capable of incubating a diversified oral microbial community in vitro [38,39]. As has been reported, the microbial communities of ECC-affected children exhibited higher metabolic activities than those of caries-free children [40]. Based on our results, the growth and metabolic activities of S-ECC saliva-derived biofilms were significantly inhibited by QAMs-modified giomers. This indicated that the novel giomers had strong antibacterial activity against S-ECC related species. We also noticed that DMAHDM-modified giomers showed comparatively better antibacterial activity than DMADDM-modified giomers, which was in good agreement with the results reported by other researchers that QAMs with longer chain length would exhibit stronger penetrating effects against bacterial membranes [41,42,43].

Furthermore, the compositions of the 72 h-biofilms on QAMs-modified giomer specimens were analyzed. Some researchers have reported an elevation of species richness in caries-affected subjects [44,45]. In our study, we found no significant difference in species richness between each QAMs group and the control group, as has been shown in the Chao index. Interestingly, however, in the PCoA analysis, the 2.5% DMADDM, 1.25% DMAHDM and 2.5% DMAHDM groups were separated from the control group, demonstrating that QAMs-modified giomers also had the potential to regulate the microbial compositions of the S-ECC saliva-derived biofilms. We found the percentages of *Streptococcus*, which has been reported to play a fundamental role in the development of ECC [46,47], significantly reduced in the 2.5% DMADDM group and the 1.25% DMAHDM group. The 2.5% DMAHDM group also showed a slightly lower level of *Streptococcus*, though it was not statistically significant. Moreover, in the 2.5% DMAHDM group, a reduced proportion of *Lactobacillus* was detected. Some studies have demonstrated that *Lactobacillus* was also related to ECC and mainly implicated in caries progression [46,48]. We also noticed that *Veillonella* was detected in a higher level in the 2.5% DMAHDM group than in the control group. *Veillonella* is a commensal microbe in the oral cavity and its role in ECC is still unclear. Some researchers found that *Veillonella* could consume lactic acids and help alleviate the demineralization of dental tissues, while others found that its consumption of acids produced by *Streptococcus* might facilitate the growth of *Streptococcus* and biofilm formation [46,47]. The elevation in the relative abundance of *Veillonella* for the 2.5% DMAHDM group might be attributed to the reduction of other genera, especially *Lactobacillus.* In addition, *Aggregatibacter* and *Haemophilus* were detected in high proportions in several samples from the DMAHDM groups, but were rarely found in other groups. As has been concluded by some researches, *Aggregatibacter* and *Haemophilus* were more abundant in caries-free children and might be associated with caries-free status [46,49]. The mechanisms inducing these microbial changes are still unclear and require further studies. In addition, we realized that since bacterial cell death in the live/dead staining of the saliva-derived biofilms, was observable at 72 h, some dead bacterial cells might be retained in the biofilms, and their DNA would be extracted along with live cells. Therefore, in the 16S rRNA sequencing, retention of dead bacteria might make it difficult to clearly identify the compositions of live bacteria in the biofilm, and might to some extent influence the sequencing results. Even so, based on the preliminary results of the present study, we prudently speculated that QAMs-modified giomers, especially DMAHDM-modified giomers, have created an environment favorable to some health-related species but disadvantageous to caries-related species. Consequently, the communities of saliva-derived biofilms would be less cariogenic. Thus, the QAMs-modified giomers exhibited great potential to regulate the compositions of cariogenic biofilms.

In the rat model, no evident inflammatory response was observed in the buccal/palatal mucosae (Figure S6), showing acceptable biocompatibility of DMADDM/DMAHDM, which has been reported previously [21,50,51]. By comparing the severity of secondary caries in the control group and in the QAMs groups, the efficacy of the QAMs-modified giomers to inhibit secondary caries was evidently demonstrated. The range and depth of carious lesions were reduced in QAMs groups, as can be reflected in the lower modified Keyes’ scores. This anti-caries ability of QAMs-modified giomers was further confirmed by the micro-CT scanning, which showed significantly lower lesion depth and mineral loss in the QAMs groups, indicating minor caries invasion and less demineralization of the dental hard tissues. Therefore, the present results implied that the novel QAMs-modified giomers have great potential in restorative treatment against ECC.

## 4. Materials and Methods

### 4.1. Fabrication of DMADDM (Dimethylaminododecyl Methacrylate)/DMAHDM (Dimethyla Minohexadecyl Methacrylate) Modified Giomers

DMADDM and DMAHDM were synthesized referring to the previous studies [52,53,54]. DMADDM/DMAHDM was then added into a commercial giomer (Beautifil II F00 A1, SHOFU Inc., Kyoto, Japan) at mass fractions of 1.25%, 2.5% and 5.0%, respectively. The commercial giomer without DMADDM/DMAHDM served as the control.

### 4.2. Mechanical Properties

A computer-controlled Universal Testing Machine (5500 R, MTS, Cary, NC, USA) was used and the three-point bending tests were carried out to measure the flexural strength and elastic modulus of bar-shaped specimens (25 mm in length, 2 mm in width and 2 mm in thickness, 6 for each group). The flexural strength was calculated by FS = 3P_max_L/(2bh^2^), while the elastic modulus was calculated by E = (P/d)(L^3^/(4bh^3^)) [55].

### 4.3. Surface Characterizations

#### 4.3.1. Surface Charge Density

A fluorescein dye method was used to measure the surface charge densities of giomer specimens [21,56]. Typically, six specimens were prepared and light-cured using a 96-well plate lid (approximately 8 mm in diameter and 0.5 mm in depth) and were tested for each group. The disks were put in a 48-well plate, added with 10 mg/mL fluorescein sodium salt in deionized (DI) water. The disks were left in the dark for 10 min at room temperature, then rinsed with DI water to remove the fluorescein solution. Thereafter the disks were transferred to a new 48-well plate to which was added 0.1% (by mass) of cetyltrimethylammonium chloride (CTMAC) in DI water, and this was shaken in the dark for 20 min at room temperatures. The bound dye was thus desorbed and the CTMAC solution was supplemented with 10% (by volume) of 100 mM phosphate buffer at pH 8. The absorbance was read at 501 nm using a microplate reader (SpectraMax M5, Molecular Devices, Sunnyvale, CA, USA). The fluorescein concentrations of the disks were calculated by Beers Law with an extinction coefficient of 77 mM^−1^ cm^−1^. The surface charge densities were then calculated.

#### 4.3.2. Contact Angle

Six specimens fabricated using a 96-well plate lid were tested for each group. A droplet of distilled water was placed on each disk and the contact angles on both sides of the droplet were measured by a drop shape analyzer (DSA100, KRÜSS, Hamburg, Germany). The contact angle value reported for each specimen was the average of the right and left contact angles.

#### 4.3.3. Surface Roughness

Specimens were prepared using a round mold (10 mm in diameter, 1 mm in depth, six for each group). The surface morphology of each specimen was evaluated by an Atomic Force Microscope (SPM-9600; Shimadzu, Kyoto, Japan) and the surface roughness was calculated over an area of 10 μm × 10 μm [57]. The surface roughness (Ra) values in different groups were then compared.

### 4.4. Colorimetric Analysis

A spectrophotometer (VITA Easyshade Advance, Vita Zahnfabrik, Bad Säckingen, Germany) was used and three random regions of each specimen (10 mm in diameter and 1 mm in depth, six for each group) were measured. The Uniform Color Space (UCS) system was used in the present study, and L* (movement in the white-black direction), a* (movement in the red-green direction), b* (movement in the yellow-blue direction) were recorded [58]. The color data of control group served as a baseline. The color difference was calculated via the equation as follows: E* = ((L*_2_ − L*_1_)^2^ + (a*_2_ − a*_1_)^2^ + (b*_2_ − b*_1_)^2^) ^1/2^, where L*_1_, a*_1_ and b*_1_ corresponded to the mean values of specimens in the control group, while L*_2_, a*_2_ and b*_2_ represented the measurements of each specimen in the other four groups.

### 4.5. Cytotoxicity Test

The specimens fabricated using a 96-well plate lid were rinsed in distilled water to remove uncured monomers and sterilized with ethylene oxide at 37 °C. Six specimens were immersed in 10 mL of Dulbecco’s Modified Eagle Medium (DMEM) with 2% fetal bovine serum, 100 IU/mL penicillin, and 100 IU/mL streptomycin, then agitated for 24 h at 37 °C to acquire the original eluants. The original eluants were serially diluted at 32-fold, 64-fold and 128-fold according to previous studies [21,59], and the specimen volume/solution volume ratios were 0.12, 0.24 and 0.47 mm^3^/mL, respectively. Human oral keratinocyte (HOK, JENNIO, Guangzhou, China) cells were inoculated in 96-well plates at 5000 cells/well with 100μL eluant-containing medium at 37 °C (5% CO_2_). After 48 h, the cell viability was finally measured by the CCK-8 (Cell Counting Kit-8, APExBIO, TX, USA) assay following the manufacturer’s instructions.

### 4.6. Fluoride Release and Recharge

Six specimens (10 mm in diameter, 1 mm in depth) were placed in 12-well plates, respectively. Each disk was dipped in 2 mL of distilled/deionized water at 37 °C for 24 h. Next, the specimens were removed and 2 mL of total ionic strength adjustment buffer II (TISAB II) was added into each well. A fluoride ion-selective electrode (7102, Fangzhou Technology, Beijing, China) was used to evaluate the fluoride releasing capacity at time intervals of one day for 21 days in total.

After the 21-day initial fluoride release, all specimens were rinsed in deionized water and dried. Fluoride recharge was carried out with a commercial fluoride varnish containing 5% sodium fluoride (NovaBright, Nanova Biomaterials Inc., Columbia, MO, USA) and the specimens were soaked for 4 min. The specimens were then rinsed with distilled/deionized water and dried before they were placed in new 12-well plates. The ion re-release after fluoride recharge was measured as described above in the following seven days.

### 4.7. Antibacterial Activity Assessment

#### 4.7.1. Saliva Sampling and Biofilm Formation

Saliva sampling was authorized by the Ethics Committee of West China Hospital of Stomatology, Sichuan University (WCHSIRB-D-2021-500). Ten preschool children diagnosed as S-ECC who had not taken any antibiotics in three months were recruited as saliva donors. Guardians of each child had signed an informed consent before saliva sampling. An equal volume of collected saliva from each subject was pooled together and diluted two-fold with sterile 50% glycerol, then stored at −80 °C [60,61].

48-well plate lid molded specimens (six for each group) were rinsed in distilled water to remove uncured monomers and sterilized with ethylene oxide at 37 °C. For saliva-derived biofilm formation, the disks were placed into 24-well plates and immersed in 1.5 mL of SHI medium [38]. The saliva-glycerol stock was then seeded (1:30 final dilution) into each well and incubated at 37 °C anaerobically (90% N_2_, 5% CO_2_, 5% H_2_). The growth medium was refreshed every 24 h.

#### 4.7.2. Lactic Acid Production by S-ECC Saliva-Derived Biofilms

24 h, 48 h and 72 h biofilms were incubated on specimen disks. Six disks were measured for each group at each time period. The disks were then washed with PBS and immersed in 1.5 mL of buffered peptone water (BPW) supplemented with 1.0% sucrose and incubated at 37 °C in 5% CO_2_ for 4 h. The lactic acid production was then measured using a lactic acid assay kit (A019-2-1, Jiancheng Bioengineering Institute, Nanjing, China) following the manufacturer’s instructions, and the absorbance at 530 nm was measured.

#### 4.7.3. MTT Assay

A MTT (3-[4,5-dimethylthiazol-2-yl]-2,5-diphenyltetrazolium bromide) assay was performed to determine the metabolic activities of S-ECC salivary derived biofilms [62]. Disks with 24 h, 48 h and 72 h biofilms (n = 6) were transferred to new 24- well plates, and each sample was incubated with 1 mL MTT dye (0.5 mg/mL MTT in PBS) for another 1 h. During this period, metabolically active bacteria would metabolize the yellow-color MTT to purple-color formazan. The samples were then transferred to new 24- well plates filled with 1 mL of dimethyl sulfoxide (DMSO), which can solubilize the formazan crystals. The plates were incubated with gentle shaking for 20 min at room temperatures. The absorbance at 540 nm was measured and compared. 

#### 4.7.4. Biofilm Imaging

Live/dead staining was carried out to observe the biofilm growth and viability. Disks with 24 h, 48 h and 72 h saliva-derived biofilms were washed with sterile water, then stained with 2.5 µM SYTO^TM^ 9 (Molecular Probes, Invitrogen, Carlsbad, CA, USA) and 2.5 µM propidium iodide (Molecular Probes, Invitrogen, Carlsbad, CA, USA) for 15 min according to the manufacturer’s instructions. A confocal laser scanning microscopy (FV3000 confocal laser scanning microscope, Olympus Corp, Tokyo, Japan) was used to acquire the images of biofilms. Live bacteria will emit a green fluorescence while dead bacteria will emit a red fluorescence.

#### 4.7.5. 16S rRNA Gene Sequencing

Six disks with 72 h-biofilms in each group were prepared for 16S rRNA gene sequencing on the Illumina MiSeq platform (Majorbio, Shanghai, China) [57]. The raw data was available in the NCBI Sequence Read Archive (SRA) database (BioProject: PRJNA808624). The microbial community analysis was performed on the Majorbio I-Sanger Cloud Platform.

### 4.8. Evaluation of Secondary Caries in a Rat Model

A rat model was used to assess the inhibition effect of QAMs-modified giomers on secondary caries by reference of a previous study [63]. In brief, specific-pathogen-free female Wistar rats (Dossy Experimental Animals Co., Ltd., Chengdu, China) at the age of 21 days were used, and after cavity preparation in their first grooves of the maxillary first molars, all cavities were restored with tested giomers used in this study. A total of 60 dental restorations from 30 rats (six for each group) were performed. After a three-day bacteria-scrubbing using a culture of *Streptococcus mutans* UA159 (10^8^ CFU/mL, 0.3 mL), all rats were orally infected. The rats then followed a cariogenic diet 2000 (Trophic Diet, Trophic Animal Feed, Suzhou, China) and drank 5% sucrose-containing water for four weeks to produce secondary caries. The rats were then sacrificed and their maxillaries were obtained. The soft tissues were fixed using 4% paraformaldehyde, then paraffin-embedded and subjected to HE staining for histopathological analysis. The restoratives of the maxillaries were removed (for better observation and dyeing) before they were subjected to a micro-CT scanning [57]. The micro-CT settings were as follows: medium resolution, with parameters 70 kVp, 200 μA, Al 0.5 mm, 1 × 300 ms, Voxel size 10 μm. A line was chosen as the region of interest (ROI) [63] for analysis. Thereafter, the maxillaries were dyed with 0.4% ammonium purpurate for 12 h, and hemi-sectioned in the mesiodistal direction and finally scored using a modified Keyes’ scoring method to evaluate the penetration of a lesion at three planes: the S-plane (slight), M-plane (moderate) and X-plane (extensive) [63]. The animal experiments were conducted following the guiding principles of the Ethics Committee of the West China Hospital of Stomatology, Sichuan University (WCHSIRB-D-2021-333).

### 4.9. Statistical Analysis

In the present study, one-way analysis of variance (ANOVA) with Tukey’s multiple comparisons was performed to compare the data. It was considered as statistically significant when the *p*-value < 0.05.

## 5. Conclusions

This study developed new giomers by incorporating DMADDM and DMAHDM into a commercial giomer. The QAMs-modified giomers provided good mechanical and surface properties, esthetic performance, cytotoxity and fluoride release. In the in-vitro S-ECC biofilm model, the new giomers exhibited excellent antibacterial activity and great potential to regulate the compositions of biofilms. DMAHDM-modified giomers showed better antibacterial activity than DMADDM-modified giomers. The modified giomers were efficient in inhibiting secondary caries in a rat model. Therefore, QAMs-modified giomers are promising as novel restorative materials in inhibiting cariogenic biofilms and secondary caries in children.

## Data Availability

The data presented in this study are available on request from the corresponding author (J.L. and J.Z.).

## Supplementary Materials

The following supporting information can be downloaded at: https://www.mdpi.com/article/10.3390/pathogens11050578/s1, Figure S1: The molecular structures of DMADDM and DMAHDM; Figure S2: Contact angle. (A–E) Representative images of the control (A), 1.25% DMADDM (B), 2.5% DMADDM (C), 1.25% DMAHDM (D) and 2.5% DMAHDM (E). Scale bars = 1 mm; Figure S3: Surface roughness. (A–E) Representative 3D images of the control (A), 1.25% DMADDM (B), 2.5% DMADDM (C), 1.25% DMAHDM (D) and 2.5% DMAHDM (E); Figure S4. Colorimetric analysis. (A–C) Values of the *L** (A), *a** (B), *b** (C). Data are presented as mean ± SD; *n* = 6. In each plot, bars with the same letter(s) indicate no significant difference between the groups (*p* > 0.05). Bars without the same letter(s) indicate significant difference between the groups (*p* < 0.05). (D) The three-dimensional scatter diagram (*L**, *a**, *b**); Figure S5: The cytotoxicty test of the eluants against HOK cells. Data are presented as mean ± SD; *n* = 6. There were no significant difference in all groups (*p* > 0.05); Figure S6: 16S r RNA sequencing. (A) Rarefaction curves. (B) Venn gram. (C,D) Proportion of *Ag**gregatibacter* (C) and *Haemophilus* (D) in each sample; Figure S7: Representative histologic images of buccal mucosae: the control (A), 1.25% DMADDM (C), 2.5% DMADDM (E), 1.25% DMAHDM (G) and 2.5% DMAHDM (I). Representative histologic images of palatal mucosae: the control (B), 1.25% DMADDM (D), 2.5% DMADDM (F), 1.25% DMAHDM (H) and 2.5% DMAHDM (J). Scale bars = 100 μm.
