# Supplementary material for: Novel Giomers Incorporated with Antibacterial Quaternary Ammonium Monomers to Inhibit Secondary Caries
Source: Pathogens. 2022 May 14;11(5):578. doi: 10.3390/pathogens11050578 (PMC9147272; doi:10.3390/pathogens11050578)
Supplement: Supplementary file 1 [file pathogens-11-00578-s001.zip › pathogens-1673353-SI/Supplementary materials (R2).pdf]

pathogens

MDPI

Supplementary Materials

Novel Giomers Incorporated with Antibacterial Quaternary Ammonium Monomers to Inhibit Secondary Caries

Yandi Chen[1,2], Bina Yang[1,3], Lei Cheng[1,3], Hockin H.K. Xu[4,5,6], Hao Li[1,3], Yuyao Huang[1,3], Qiong Zhang[1,2], Xuedong Zhou[1,3], Jingou Liang[1,2,*] and Jing Zou[1,2,*]

1 State Key Laboratory of Oral Diseases & National Clinical Research Center for Oral Diseases, West China School of Stomatology, Sichuan University, Chengdu 610041, China; chenyandi1992@163.com (Y.C.); 386908361@qq.com (B.Y.); chenglei@scu.edu.cn (L.C.); 529423563@qq.com (H.L.); huangyuyaott@163.com (Y.H.); zhangqiongdentist@126.com (Q.Z.); zhouxd@scu.edu.cn (X.Z.)
2 Department of Pediatric Dentistry, West China School of Stomatology, Sichuan University, Chengdu 610041, China
3 Department of Cariology and Endodontics, West China School of Stomatology, Sichuan University, Chengdu 610041, China
4 Department of Advanced Oral Sciences and Therapeutics, School of Dentistry, University of Maryland, Baltimore, MD 21201, USA; Hxu@umaryland.edu (H.H.K.X.).
5 Center for Stem Cell Biology and Regenerative Medicine, School of Medicine, University of Maryland, Baltimore, MD 21201, USA
6 Marlene and Stewart Greenebaum Cancer Center, School of Medicine, University of Maryland, Baltimore, MD 21201, USA
* Correspondence: liangjingoudentist@163.com (J.L.); zoujing@scu.edu.cn (J.Z.).

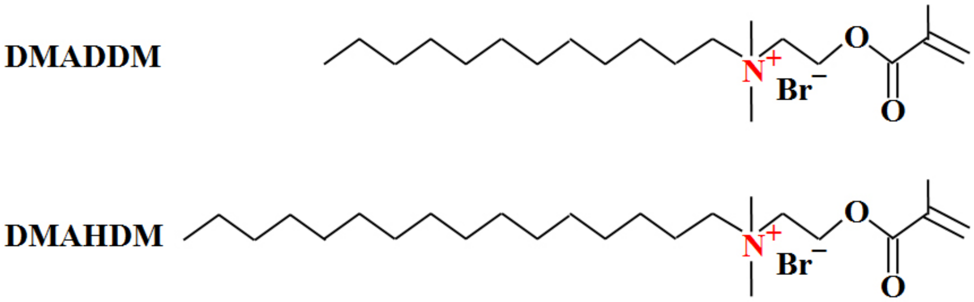

Figure S1. The molecular structures of DMADDM and DMAHDM.

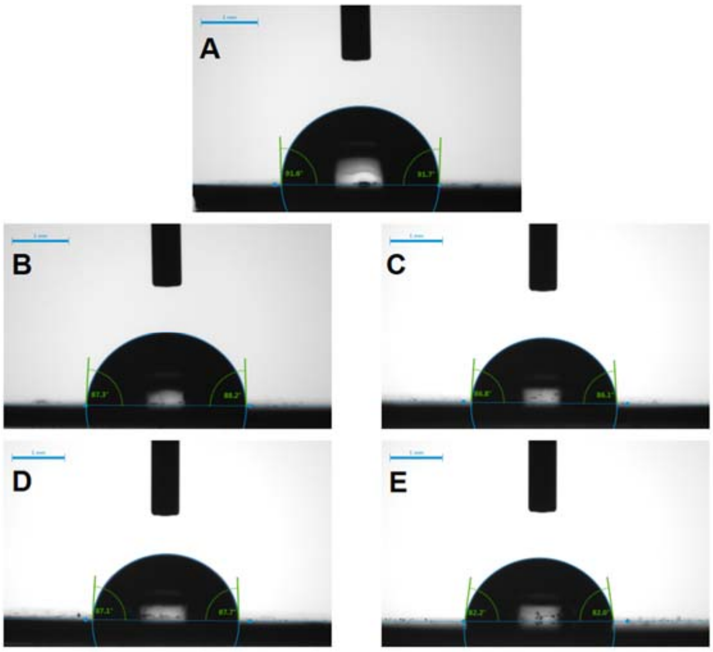

Figure S2. Contact angle. (A-E) Representative images of the control (A), 1.25% DMADDM (B), 2.5% DMADDM (C), 1.25% DMAHDM (D) and 2.5% DMAHDM (E). Scale bars = 1 mm.

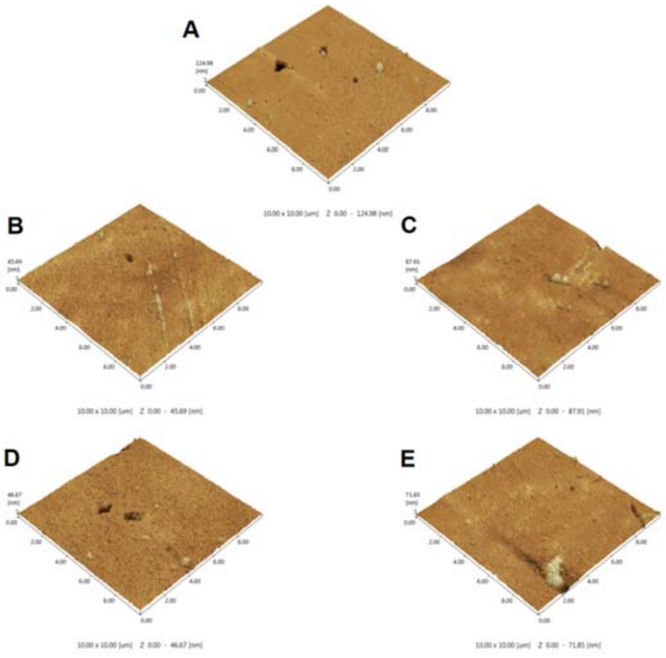

Figure S3. Surface roughness. (A-E) Representative 3D images of the control (A), 1.25% DMADDM (B), 2.5% DMADDM (C), 1.25% DMAHDM (D) and 2.5% DMAHDM (E).

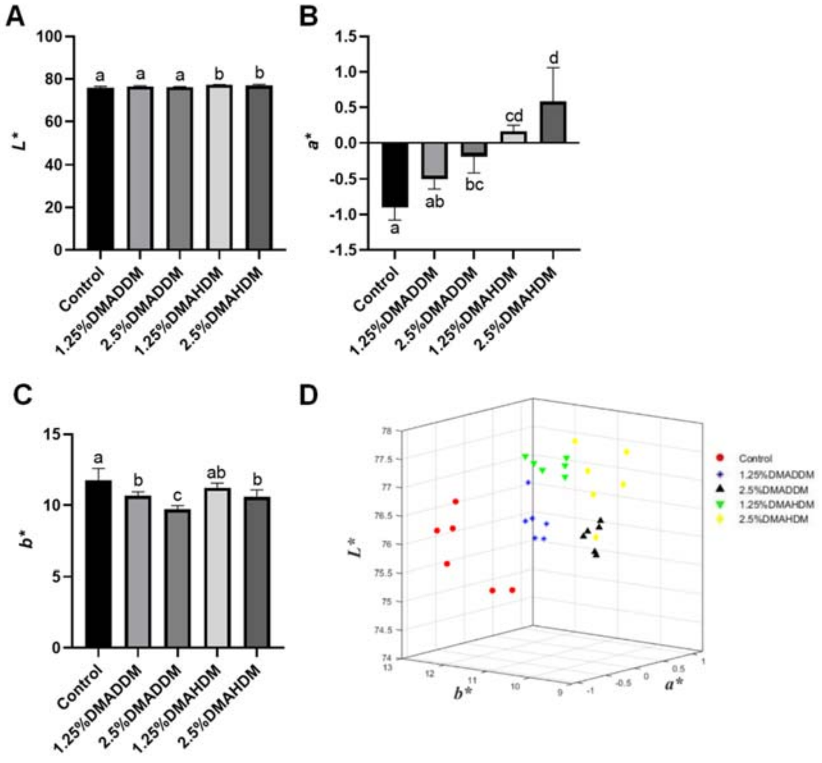

Figure S4. Colorimetric analysis. (A-C) Values of the L^* (A), a^* (B), b^* (C). Data are presented as mean ± SD; n = 6. In each plot, bars with the same letter(s) indicate no significant difference between the groups ($p > 0.05$). Bars without the same letter(s) indicate significant difference between the groups ($p < 0.05$). (D) The three-dimensional scatter diagram (L^*, a^*, b^*).

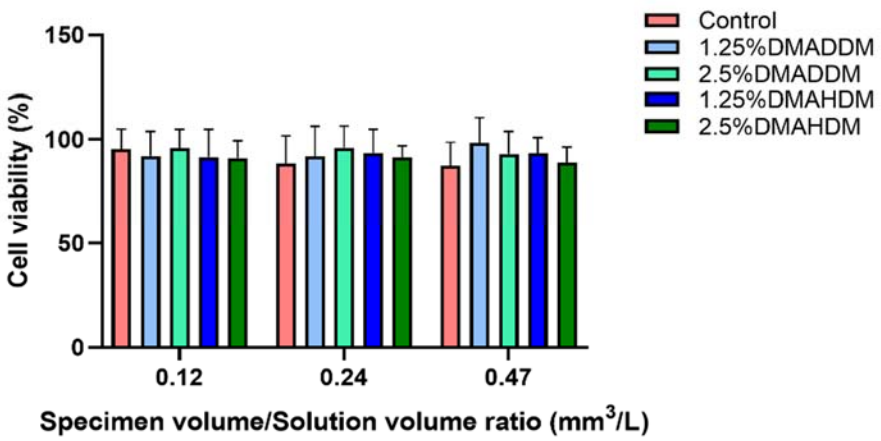

Figure S5. The cytotoxicty test of the eluants against HOK cells. Data are presented as mean ± SD; n = 6. There were no significant difference in all groups ($p > 0.05$).

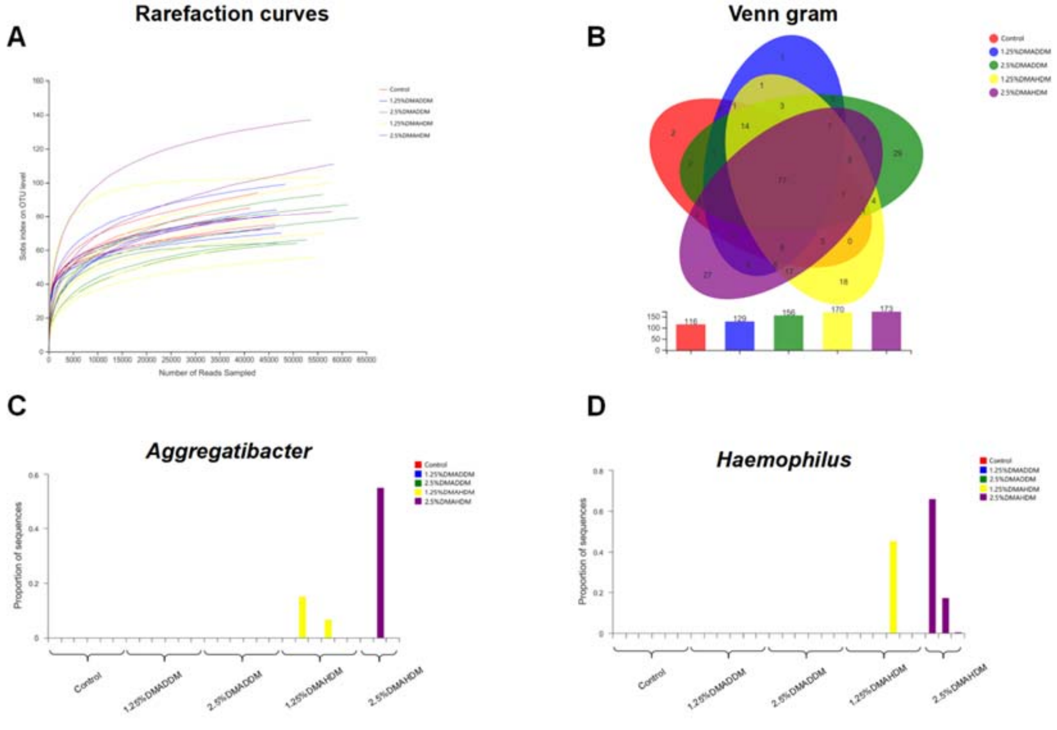

Figure S6. 16S r RNA sequencing. (A) Rarefaction curves. (B) Venn gram. (C,D) Proportion of *Aggregatibacter* (C) and *Haemophilus* (D) in each sample.

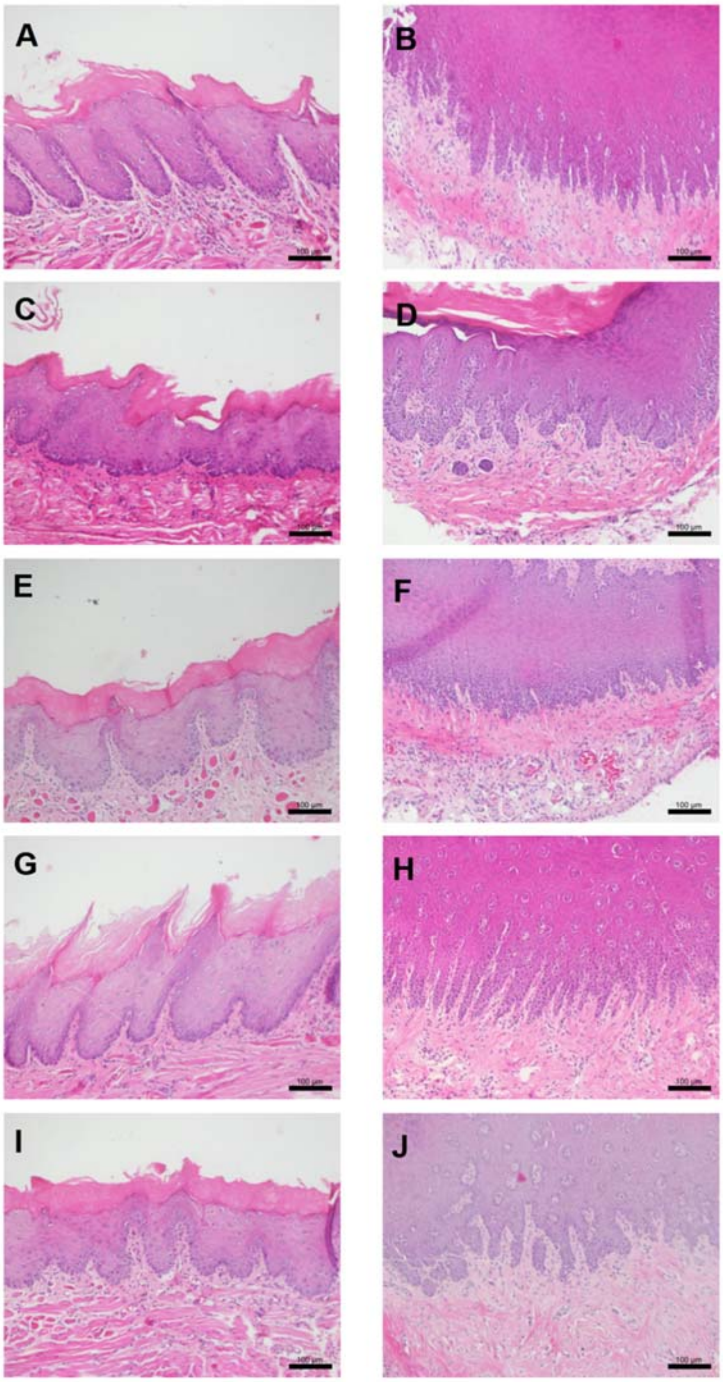

Figure S7. Representative histologic images of buccal mucosae: the control (A), 1.25% DMADDM (C), 2.5% DMADDM (E), 1.25% DMAHDM (G) and 2.5% DMAHDM (I). Representative histologic images of palatal mucosae: the control (B), 1.25% DMADDM (D), 2.5% DMADDM (F), 1.25% DMAHDM (H) and 2.5% DMAHDM (J). Scale bars = 100 μm.
